# Chromosome-level genome assembly of *Plagiognathops microlepis* based on PacBio HiFi and Hi-C sequencing

**DOI:** 10.1038/s41597-024-03645-x

**Published:** 2024-07-19

**Authors:** Yidi Wu, Hang Sha, Xiangzhong Luo, Guiwei Zou, Hongwei Liang

**Affiliations:** 1 Yangtze River Fisheries Research Institute, Chinese Academy of Fishery Sciences, Wuhan, 430223 China; 2 Key Laboratory of Aquatic Genomics, Ministry of Agriculture and Rural Affairs, Yangtze River Fisheries Research Institute, Chinese Academy of Fishery Sciences, Wuhan, 430223 China

**Keywords:** Genome, Genomics

## Abstract

*Plagiognathops microlepis* is an economic freshwater fish in the subfamily *Xenocyprinae* of Cyprinidae. It is widely distributed in the freshwater ecosystem of China, with moderate economic value and broad development prospects. However, the lack of genomic resources has limited our understanding on the genetic basis, phylogenetic status and adaptive evolution strategies of this fish. Here, we assembled a chromosome-level reference genome of *P. microlepis* by integrating Pacbio HiFi long-reads, Illumina short-reads and Hi-C sequencing data. The size of this genome is 1004.34 Mb with a contig N50 of 38.80 Mb. Using Hi-C sequencing data, 99.59% of the assembled sequences were further anchored to 24 chromosomes. A total of 578.91 Mb repeat sequences and 28,337 protein-coding genes were predicted in the current genome, of which, 26,929 genes were functionally annotated. This genome provides valuable information for investigating the phylogeny and evolutionary history of cyprinid fishes, as well as the genetic basis of adaptive strategies and special traits in *P. microlepis*.

## Background & Summary

The smallscale yellowfin, *Plagiognathops microlepis*, belongs to the cyprinid subfamily *Xenocyprinae*. It is a small to medium-sized fish that inhabits the middle-to-bottom layers of the water and is widely distributed in freshwater ecosystems of China^1^. This fish feeds on humus, organic debris and algae, therefore it is often used as a tool fish to purify water and control algal bloom, playing an important role in freshwater systems^2,3^. With the advantages of delicious taste, fast growth and few diseases, *P. microlepis* has been domesticated into an aquaculture variety^4–6^. Due to its unique dietary characteristics, when co-cultured with other fish species, *P. microlepis* does not affect the growth of its companions. Instead, it can help to increase yield and purify water, showing broad development prospects^7,8^. In recent years, the artificial seedling breeding and stock enhancement and releasing of *P. microlepis* have been carried out in many regions of China^9^. Studies on the culture technique and models^7^, population structure^10^, nutritional composition^8^, biochemistry and toxicology^2,3^, and the roles in water quality improvement of this fish^11^ have also been conducted. However, due to the lack of genetic data resources, researches on the evolutionary adaptation strategies and the molecular mechanisms of excellent traits in *P. microlepis* are still scarce, which limit our understanding and effective utilization of this fish.

In phylogenetic studies, the subfamily *Xenocyprinae* is a branch of Cyprinidae with fewer species. These species distribute widely and discretely in East Asia (especially in China) and have a long history, making it possible to evaluate species and populations differentiation under historical environment changes in East Asia^12^. Currently, the widely accepted classification view is that this subfamily includes 10 species in 4 genera^13^. However, different views have emerged on the phylogenetic positions and evolutionary history of some fish within this subfamily as phylogenetic studies increasing. Some studies considered *P. microlepis* as the only one species in *Plagiognathops*^1,14^, while others suggest that *Plagiognathops* is not a valid genus and this fish should be classified into the genus *Xenocypris*^13,15^. Both views have been supported by phylogenetic evidences based on different molecular markers^16^. Nevertheless, most of these studies were conducted based on mitochondrial or several nuclear genes, resulting in inconsistent results. With the development of sequencing technology, phylogenetic studies have entered the era of omics. However, in the subfamily *Xenocyprinae*, only the genome of *Pseudobrama simoni* has recently been deciphered^17^. Therefore, to provide a high-quality genome of *P. microlepis* is also essential for conducting phylogenetic analysis at the genomics level and elucidating the controversy in the validity of the genus *Plagiognathops*.

In this work, we constructed a chromosome-level reference genome of *P. microlepis* by integrating 33.62 Gb of HiFi reads, 44.58 Gb of short reads, and 99.57 Gb of Hi-C reads. The assembled size of this genome was 1004.34 Mb, and 1004.12 Mb of which was anchored to 24 chromosomes with a contig N50 length of 39.98 Mb. This genome contains about 57.64% (578.91 Mb) of repeat elements and 26,929 protein-coding genes. In addition, we also assembled two chromosome-level haplotypes, Haploid A (997.69 Mb) and Haploid B (995.24 Mb), with contig N50 lengths of 36.21 Mb and 33.97 Mb respectively. The completeness (>97.0% complete BUSCO), consistency (>99.8% mapping ratio) and consensus quality values (>47.5) of all the 3 assemblies were estimated to be high. Overall, this genome will provide a reference for phylogenetic, adaptative evolutionary and genetic basis studies on *P. microlepis* and other cyprinid fishes, which may also provide valuable information for the regulation and restoration of freshwater ecosystems. And the assembled haplotype genomes can serve as a baseline for studies on allele-specific expression or conservation genomics.

## Methods

### Ethics statement

This work was approved by the Care and Use of Laboratory Animals in Yangtze River Fisheries Research Institute, Chinese Academy of Fishery Sciences (Wuhan, China).

### Sampling and genome survey

**Fig. 1:**
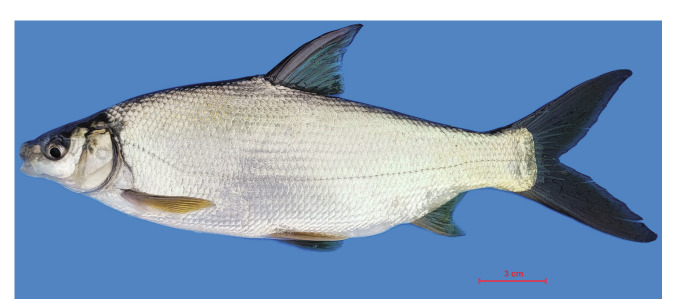
Morphological characters of the *Plagiognathops microlepis* used for genome sequencing.

A healthy female *P. microlepis* (body weight: 539.79 g) was collected from the original breeding farm in Suizhou, Hubei Province, China (Fig. 1). After anesthesia with MS222 (0.05% in concertation), the muscle, heart, liver, brain, gill and spleen tissues were immediately sampled and frozen in liquid nitrogen, then transferred to −80 °C for further use. High-quality genomic DNA (gDNA) of muscle was extracted with a modified cetyltrimethyl ammonium bromide (CTAB) method^18^, while the total RNAs were isolated from all tissues using the Omega Bio-tek’s E.Z.N.A.^®^ Total RNA Kit I (R6834, Omega, USA). The quality and concentration of DNA (and RNA) were tested by 0.75% (and 1.5%) agarose gel electrophoresis, NanoDrop One spectrophotometer (Thermo Fisher Scientific) and Qubit 3.0 Fluorometer (Life Technologies, Carlsbad, CA, USA).

For genome survey, libraries with 350 bp insert size were constructed using the Nextera DNA Flex Library Prep Kit (Illumina, San Diego, CA, USA) and sequenced with PE-150 paired-end strategy on Illumina Novaseq 6000 platform. After obtaining raw data (44.58 Gb), the sequencing adaptors and low-quality reads were filtered using the Fastp (v 0.21.0) tool. The 19-mer frequency depth distribution was constructed using Jellyfish (v 2.2.10)^19^, and the genome size was subsequently estimated with Jellyfish and Genomescope (v 2.0)^20^. Finally, based on the obtained 43.72 Gb clean data, the estimated genome size for *P. microlepis* was 949.39 Mb with a heterozygous ratio of 0.52 (Fig. S1).

### PacBio and Hi-C based whole-genome sequencing

For PacBio sequencing, the qualified high-quality gDNA was sheared to 15–20 Kbp and a SMART bell library was constructed according to the manufacturer’s instructions, followed by sequenced on PacBio Sequel II system in CCS mode. Raw subreads were obtained after filtering polymerase reads, and then processed using SMARTLink (v 8.0) with the parameter “–min-passes = 3–min-rq = 0.99” to generate HiFi reads. As a result, a total of 33.62 Gb HiFi reads were obtained with a mean read length of 17.82 Kb (Table 1).

**Table 1:** Summary of the sequencing data used in the assembly and annotation of *Plagiognathops microlepis* genome.

Library types	Sample	Platform	Bases (Gb)	Reads Count	Mean length (bp)	N50 (bp)
SMRT Bell	muscle	PacBio Sequel II (HiFi)	33.62	1,886,766	17,821	17,008
Hi-C	muscle	Illumina Novaseq 6000	99.57	663,833,500	150	150
Short-read	muscle	Illumina Novaseq 6000	44.58	297,234,724	150	150
RNA-seq	mix	Illumina Novaseq 6000	11.48	38,269,877	150	150

Note: Sample used for RNA-seq are mix of the muscle, heart, liver, brain, gill and spleen tissues.

A Hi-C library was constructed by cross-linking the muscle tissue, digesting with *Dpn II* restriction enzyme, biotinylating 5′ overhang, blunt-end ligation, and shearing the DNA into 300–700 bp size^21^. Hi-C sequencing was performed on Illumina Novaseq 6000 platform with PE-150 strategy, and 99.57 Gb of raw data were obtained. After filtering with fastp (v 0.21.0), 98.31 Gb of Hi-C clean reads were retained (Table 1). All of the above sequencing was performed in the Wuhan Benagen Technology Co., Ltd (Wuhan, China).

### *De novo* assembly and Hi-C assembly

In order to generate the monoploid and two haplotype-resolved assembly, the Hi-C integration strategy was used for *de novo* assembly. HiFi long reads and Hi-C short reads were submitted simultaneously to HiFiasm (v 0.16.1)^22^ to improve the accuracy of assembly and haplotype construction. Haplotypic duplications in the assembly were removed using purge_dups (v1.2.5, parameter: -f 0.9)^23^. As a result, a preliminary monoploid assembly of 1004.34 Mb and two haploid assemblies of 997.69 Mb (Haploid A) and 995.24 Mb (Haploid B) were yielded, whose contig N50 lengths were 38.80 Mb, 36.21 Mb and 33.97 Mb, respectively (Table 2). The size of this genome is slightly larger than that of survey result and the previously assembled genome of *P. simoni*’s (940.9 Mb) in *Xenocyprinae*.

**Table 2:** Statistics and evaluation of the genome assemblies of monoploid and two haploids.

	Genome	Haploid A	Haploid B
Total length (bp)	1,004,342,920	997,689,109	995,239,663
GC content (%)	37.46	37.45	37.44
Contig number	35	55	48
Contig N50 (bp)	38,802,614	36,213,105	33,971,167
Contig N90 (bp)	31,324,152	13,102,075	15,549,623
Average contig length (bp)	28,695,512	18,139,801.98	20,734,159.65
Maximum contig length (bp)	56,589,079	51,287,089	53,510,343
Scaffold number	29	33	32
Scaffold N50 (bp)	39,984,115	39,350,168	38,591,496
Scaffold N90 (bp)	33,350,185	33,143,256	33,199,758
Average scaffold length (bp)	34,632,535.17	30,233,069.97	31,101,289.47
Maximum scaffold length (bp)	70,153,910	70,172,539	71,079,337

**Fig. 2:**
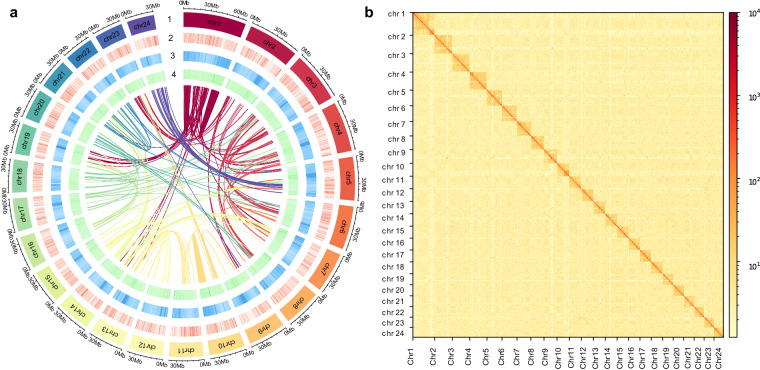
Circos plot (**a**) and Hi-C interaction heatmap (**b**) showing the features and interactions among chromosomes of the assembled *P. microlepis* genome. Tracks from outer to inner layers represent the 24 chromosomes (1), gene density (2), repeat elements density (3), GC content (4) and links of intragenomic syntenic blocks within 100Kbp sliding windows.

For the chromosome-level assembly, clean Hi-C reads were mapped to the preliminary assembled genome and filtered using Jucier (v1.6)^24^ with default parameters. Only valid interaction pairs were retained for further analysis. As a diploid, the chromosome number of *P. microlepis* is reported to be 2n = 48 based on karyotype analysis^9,25,26^. Therefore, we used the software 3D-DNA (v 180419)^27^ and Jucier to scaffold the genome onto 24 chromosomes, followed by using Juicebox (v 1.11.08)^28^ to manually adjust and orient the chromosomes and draw Hi-C interaction heatmap of contigs. Ultimately, 99.98% and 99.59% of the preliminary assembled contigs were anchored to the chromosomes of monoploid (24 chromosomes) and two haplotypes (48 chromosomes) respectively, and a chromosome-level genome of *P. microlepis* with haplotype-resolution was therefore obtained (Fig. 2, S2 and Table 3). In the assembled monoploid and two haplotypes, 19 and 24 chromosomes were found gap-free (contained only one contig for each chromosome).

**Table 3:** Statistics of the 24 anchored chromosomes of monoploid and two haploids.

	Genome		Haploid A		Haploid B	
	Length (bp)	Contig number	Length (bp)	Contig number	Length (bp)	Contig number
Chr1	70,153,910	2	70,172,539	4	71,079,337	2
Chr2	57,305,270	3	55,137,214	2	55,478,754	3
Chr3	56,589,079	1	56,111,778	2	55,198,144	2
Chr4	55,730,263	1	55,624,499	3	53,510,343	1
Chr5	48,498,026	1	48,468,750	1	46,904,425	2
Chr6	48,350,784	2	48,127,576	4	48,778,166	2
Chr7	44,878,238	2	44,547,026	6	44,565,349	3
Chr8	42,428,409	1	40,375,467	2	42,263,062	1
Chr9	42,102,337	1	41,857,314	1	37,739,588	2
Chr10	39,984,115	2	39,350,168	2	39,344,695	1
Chr11	38,889,763	1	38,708,038	1	38,591,496	1
Chr12	38,802,614	1	38,180,736	1	38,723,181	2
Chr13	38,650,511	1	38,473,907	1	38,561,032	2
Chr14	38,120,063	1	38,121,869	1	38,066,241	2
Chr15	37,063,829	1	36,974,208	1	36,928,740	2
Chr16	36,687,744	1	36,555,832	3	36,489,999	2
Chr17	36,612,228	1	36,324,304	1	36,276,649	1
Chr18	36,583,452	1	36,415,455	1	36,324,138	1
Chr19	34,210,483	1	33,950,902	1	33,971,167	1
Chr20	34,157,710	1	34,085,846	1	33,847,933	1
Chr21	33,350,185	1	33,143,256	1	33,199,758	3
Chr22	32,557,549	1	32,224,051	1	32,499,267	1
Chr23	31,783,384	1	31,417,961	3	31,033,760	1
Chr24	30,628,876	1	30,646,351	2	30,443,648	1

The mitochondrial genome of *P. microlepis* was also assembled using MitoZ (v 3.6) and Getorganelle (v 1.7.1a) based on the short reads. The obtained circular mitochondrial genome is 11,619 bp in size, with 13 protein-coding genes, 22 tRNAs and 2 rRNAs (Fig. S3).

### Genome annotation

**Fig. 3:**
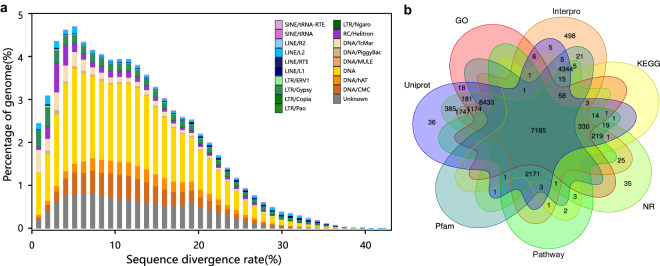
The repeat elements distribution and identified protein-coding genes in *P. microlepis* genome. (**a**) Distribution of divergence rate for transposable elements in the genome. (**b**) Veen diagram showing the number of shared and unique genes annotated with different databases.

For repeat annotation of the *P. microlepis* genome, *de novo* prediction was firstly conducted using RepeatModeler (v 1.0.11, parameters: BuildDatabase -name mydb, RepeatModeler -database mydb -pa 10)^29^ to detect repetitive elements. LTR sequences were predicted and deduplicated with LTR_FINDER_parallel (parameters: -threads 16 -harvest_out -size 1000000 -time 300) and LTR_retriever (v 2.9.0). These *de novo* predicted sequences were merged with the RepBase library (v 20181026), and RepeatMasker (v 4.0.9, parameters: -nolow -no_is -norna -parallel 2) and RepeatProteinMask (v 4.0.9) were subsequently employed to identify repeat elements and TE_protein class repeat sequences^30^. Finally, all the predicted results were merged together and deduplicated, and 578.91 Mb of repeat sequences were identified, accounting for 57.64% of the assembled genome. The most abundant element among these repetitive sequences was DNA transposon, encompassed 31.55% (316.89 Mb) of the assembled genome (Table 4 and Fig. 3a).

**Table 4:** Summary of the transposable elements in *P. microlepis* genome.

Type	TE protiens		*De novo* + repbase		Combined TEs	
	Length (bp)	% in genome	Length (bp)	% in genome	Length (bp)	% in genome
DNA	21,246,360	2.12	314,296,414	31.29	316,891,032	31.55
LINE	16,746,855	1.67	31,837,184	3.17	34,416,502	3.43
SINE	0	0	1,574,247	0.16	1,574,247	0.16
LTR	25,129,588	2.5	114,807,756	11.43	117,670,364	11.72
Satellite	0	0	6,563,915	0.65	6,563,915	0.65
Simple repeat	0	0	2,058,194	0.2	2,058,194	0.2
Other	0	0	5,172	0	5,172	0
Unknown	2,040	0	121,082,123	12.06	121,084,163	12.06
Total	63,103,527	6.28	559,942,334	55.75	578,905,265	57.64

Three prediction methods were used for the structural annotation of protein-coding genes, including transcript mapping, *ab initio* prediction and homologous gene alignment. In the RNA-seq based method, 2 μg of qualified RNA from each tissue was equally pooled and an RNA-seq library was prepared with NEBNext^®^ Ultra™ RNA Library Prep Kit (#E7530L, NEB, USA). The constructed library was sequenced on the Illumina Novaseq 6000 platform to obtain 150 bp paired-end reads. After sequencing, the obtained data (11.48 Gb) were filtered with Fastp (v0.21.0), aligned against the genome with Hisat2 (v2.1.0)^31^ and assembled with Stringtie (v2.1.4)^32^. The assembled transcripts ORF were predicted using TransDecoder (v 5.1.0). The *ab initio* prediction was conducted using Augustus (v3.3.2, parameter: –uniqueGeneId = true–noInFrameStop = true–gff3 = on–strand = both)^33^, Genscan (v1.0)^34^ and GlimmerHMM (v3.0.4, parameter: -f -g)^35^ after repeat elements were masked from the genome. In the homology-based prediction, the protein sequences of *Danio rerio* (GCF_000002035.6), *Ctenopharyngodon idellus* (GCF_019924925.1), *Megalobrama amblycephala* (GCF_018812025.1), *P. simoni*^17^ and *Hypophthalmichthys molitrix* (unpublished data, provided by our lab) were downloaded from NCBI database or GigaDB (http://GigaDB.org) and mapped to the genome using tblastn (v 2.7.1, parameter: -t 16 -q 7). Transcripts and coding regions in *P. microlepis* genome were then predicted using Exonerate (v 2.4.0, parameter: -model protein2genome -showtargetgff 1)^36^. Finally, all the gene sets predicted by three methods were integrated using MAKER (v 2.31.10, parameter: maker_exe.ctl maker_opts.ctl maker_bopts.ctl -ignore_nfs_tmp -fix_nucleotides)^37^, and incomplete genes and genes with too short CDS (<150 bp) were also removed, resulting in a non-redundant reference gene set including 28,337 protein-coding genes. The average exon number, exon length and CDS length in each gene were 9.35, 300.06 bp and 1,644.51 bp, respectively (Table 5).

**Table 5:** The annotated genes and features based on different methods.

Method	Gene set	Gene number	Average gene length (bp)	Average CDS length (bp)	Average exon per gene	Average exon length (bp)	Average intron length (bp)
*Ab initio*	Genscan	34,964	18,899.64	1,566.29	7.88	198.85	2,520.56
	AUGUSTUS	10,864	59,663.70	4,235.10	24.5	172.83	2,358.19
Homology-based	*P. simoni*	52,349	19,167.47	933.18	5.22	178.64	4,317.15
	*D. rerio*	56,139	30,121.38	1,200.72	6.07	197.81	5,704.27
	*M. amblycephala*	64,633	27,300.13	1,135.41	5.78	196.59	5,478.79
	*H. molitrix*	66,238	26,858.04	947.82	4.92	192.54	6,605.22
	*C. idellus*	54,785	30,140.00	1,230.10	6.4	192.07	5,349.31
RNAseq		20,350	20,499.20	1,545.44	9.63	342.32	1,992.33
Integration		23,997	25,428.66	2,069.13	11.13	256.98	2,227.28
Final set		28,337	20,087.81	1,644.51	9.35	300.06	2,068.92

Functional annotation of protein-coding genes was carried out by aligning the predicted sequences against entries in Uniprot and NR databases using Diamond (v 2.0.11.149, parameter: -e-value 1e-5)^38^. Gene motifs and domains were searched using InterProScan (v 5.52–86.0, parameter: -goterms -pa -dp -verbose -cpu 20)^39^ and Hmmscan (v 3.3.2, parameter: -E 0.01). The GO terms for genes were obtained from the corresponding InterPro or Uniprot entry. Pathway annotation was performed using Diamond and KOBAS (v3.0) against the KEGG database. At last, a total of 26,929 protein-coding genes were functionally annotated, representing 95.03% of all the predicted genes (Fig. 3b, Table 6).

**Table 6:** Summary for genome function annotation based on different databases.

Item	Annotated number of putative genes	Percentage
KEGG	10,010	35.32%
Pathway	9,933	35.05%
Nr	26,330	92.92%
Uniprot	26,338	92.95%
GO	17,385	61.35%
KOG	534	1.88%
Pfam	22,224	78.43%
Interpro	26,232	92.57%
Annotated	26,929	95.03%
Predicted genes	28,337	

For non-coding RNA annotation, tRNAs in the genome were searched using tRNAscan-SE (v 2.0.12, parameters: -E -j tRNA.gff -o tRNA.result -f tRNA.struct -thread 16)^40^ based on the structural characteristics of tRNA, rRNAs were predicted using RNAmmer (v 1.2, parameters: -S euk -m tsu,lsu,ssu)^41^, and ncRNA sequences were searched using INFERNAL (v 1.1.4, parameter: -cut_ga -rfam -nohmmonly -fmt 2)^42^ based on the Rfam database. Ultimately, 2,442 miRNAs, 9,677 tRNAs, 4,455 rRNAs and 1,058 snRNAs were identified (Table 7). Based on the annotation results, syntenic blocks among the 24 chromosomes were identified using MCScanX (https://github.com/wyp1125/MCScanx, parameter: -a -e 1e-5 -s 5), and a circular diagram showing the distribution of gene and repeat density, GC content and synteny in the genome was generated using circlize package^43^ (Fig. 2a).

**Table 7:** Statistic of the annotated non-coding RNAs in the genome.

Type		Copy	Average length(bp)	Total length(bp)	% of genome
miRNA		2,442	72	175,788	0.0175
tRNA		9,677	76	734,733	0.0732
rRNA	rRNA	4,455	150	669,710	0.0667
	18 S	19	1,855	35,240	0.0035
	28 S	16	7,412	118,585	0.0118
	5.8 S	1,977	115	227,592	0.0227
	5 S	2,443	118	288,293	0.0287
snRNA	snRNA	1,058	154	162,753	0.0162
	CD-box	442	178	78,716	0.0078
	HACA-box	76	152	11,555	0.0012
	splicing	517	135	69,686	0.0069
	scaRNA	9	225	2,025	0.0002

### Chromosomal synteny analysis

**Fig. 4:**
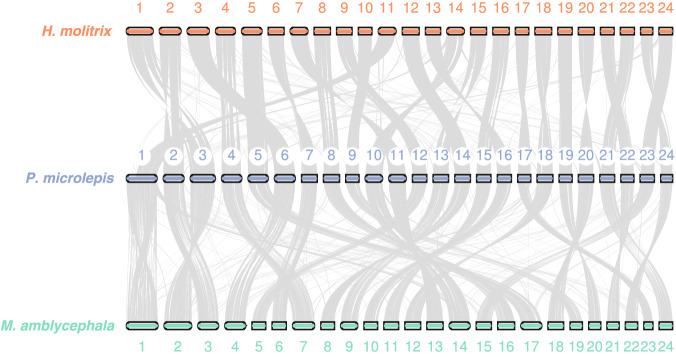
Genomic synteny among *P. microlepis*, *Megalobrama amblycephala* and *Hypophthalmichthys molitrix*.

To compare the structural characteristics of the genomes, as well as to verify the accuracy of our assemblies, genomic synteny analysis was performed between *P. microlepis* and two related species, *H. molitrix* and *M. amblycephala*. Similar gene pairs and syntenic blocks between any two of the genomes were determined and visualized using Last (v1170)^44^ and JCVI (v0.9.13)^45^. The result showed a high degree of collinearity across the three genomes, in which the structures of most chromosomes in *P. microlepis* remained unchanged compared with those of *H. molitrix* and *M. amblycephala* (Fig. 4). Such high consistency also indicated the high quality of our assembled and annotated genome.

## Data Records

The raw sequencing data reported in this paper have been deposited in NCBI Sequence Read Archive (SRA) database under the accession number SRR27884027^46^, SRR27884028^47^, SRR27884029^48^ and SRR27884030^49^. The assembled nuclear and mitochondrial genomes are available in GenBank with the accessions GCA_040144785.1^50^ and PP836169.1^51^. The genome annotation results have been deposited in the *figshare* database^52^.

## Technical Validation

### Quality evaluation of the genome assembly and annotation

Completeness of the assembled genome was evaluated using BUSCO (v 5.3.0, parameter: -m prot -c 40 -long -f)^53^ with actinopterygii_odb10 database, and 97.6%, 97.6% and 97.3% complete BUSCOs were found in the assembled monoploid, Haploid A and Haploid B genomes (Table 8), manifesting high completeness of our assembled genomes. The consistency was estimated by mapping the Illumina short-reads to the assembled genomes using BWA (v 0.7.17). As a result, high mapping rates against the three assemblies (99.87%~99.88%) and high coverages (>99.94%) were also found (Table 8). Using Merqury^54^, the consensus quality value (QV) of genomes representing per-base consensus accuracy were estimated to be 48.11, 48.01 and 47.93 for the assembled monoploid, haploid A and haploid B genomes, respectively. For the quality evaluation of chromosomes, strong interactive signals were found along the diagonals of Hi-C heatmaps, and no obvious noises were found at other areas (Fig. 2b and S2), supporting the precision of chromosome assembly. Finally, to verify the accuracy of haploid splitting, the assembled Haploid A was aligned to Haploid B and similar syntenic blocks between them were also shown. The result showed high similarity and high synteny between the two haploids, indicating high splitting accuracy (Fig. S4).

**Table 8:** Completeness and accuracy evaluation of the genome and annotation.

Type	Genome		Haploid A		Haploid B		Annotation	
	Number	Percentage (%)	Number	Percentage (%)	Number	Percentage (%)	Proteins	Percentage (%)
Complete BUSCOs (C)	3,555	97.6	3,550	97.6	3,542	97.3	3,458	95
Single-Copy BUSCOs (S)	3,506	96.3	3,500	96.2	3,496	96	3,405	93.5
Duplicated BUSCOs (D)	49	1.3	50	1.4	46	1.3	53	1.5
Fragmented BUSCOs (F)	8	0.2	11	0.3	9	0.2	46	1.3
Missing BUSCOs (M)	77	2.2	79	2.1	89	2.5	136	3.7
Total BUSCOs	3,640	100	3,640	100	3,640	100	3,640	100
Shore-reads mapping rate	—	99.88	—	99.87	—	99.87	—	—
QV	48.11	—	48.01	—	47.93	—	—	—

BUSCO analysis was also performed to validate the quality of genome annotation, and the result revealed that 95% of the identified BUSCOs (including 93.5% single-copy and 1.5% duplicated genes) were complete (Table 8). In addition, the length distributions of genes, CDSs, introns and exons in the genomes of *P. microlepis*, *D. rerio*, *P. simoni*, *H. molitrix*, *M. amblycephala* and *C. idellus* were also compared and found to be similar (Fig. S5), indicating the reliability of our genome annotation.

### Supplementary information

Supplementary Figures

## Data Availability

No custom codes were used in this study. All commands and pipelines used in data processing were executed according to the manual and protocols of the corresponding software. The parameters for different software and tools were specified in the Methods section, and default parameters were used for those without detailed parameters.
