# Supplementary material for: Chromosome-level genome assembly of Plagiognathops microlepis based on PacBio HiFi and Hi-C sequencing
Source: Sci Data. 2024 Jul 19;11:802. doi: 10.1038/s41597-024-03645-x (PMC11271555; doi:10.1038/s41597-024-03645-x)

Figure S1. The 19-mer distribution and genome size estimation of *P. microlepis*.

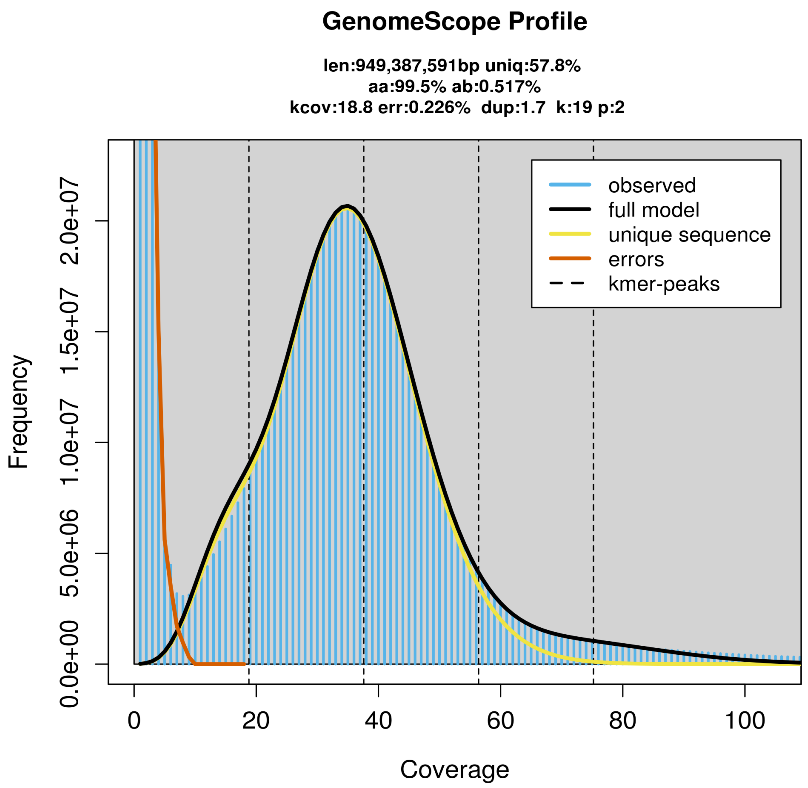

Figure S2. Hi-C interaction heatmaps of the assembled Haploid A (a) and Haploid B (b).

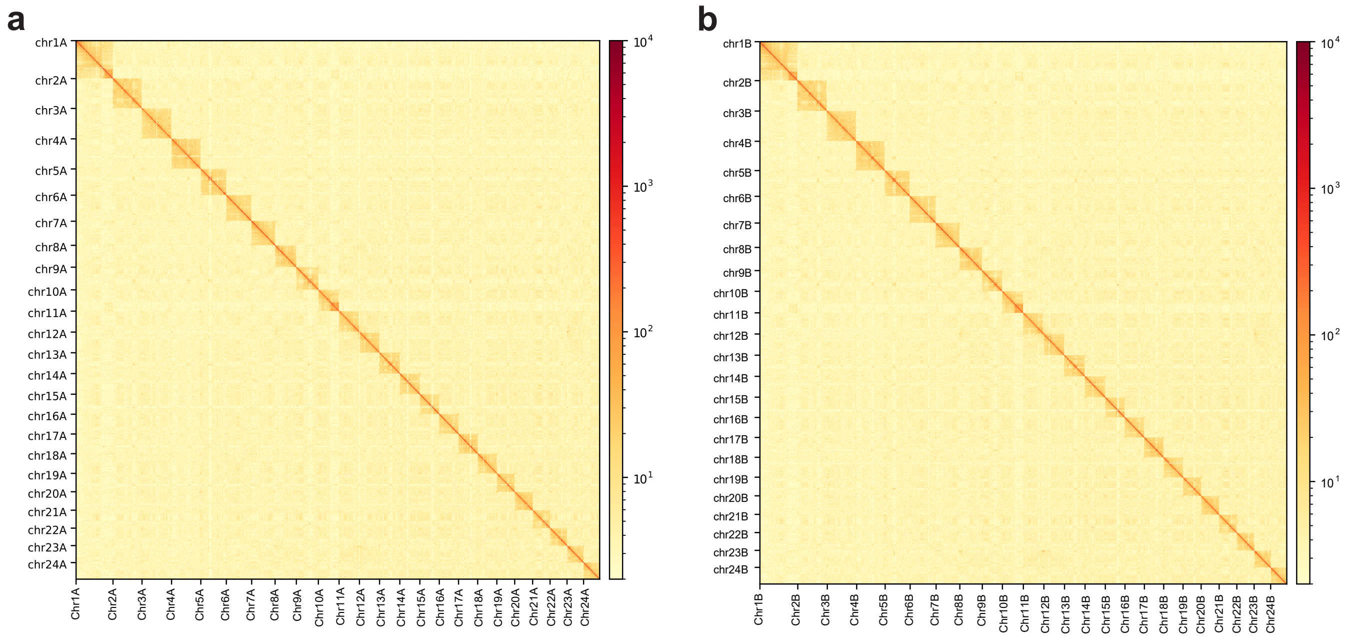

Figure S3. Distribution of annotated genes on mitochondrial genome. The inner ring shows the GC content, and the circle inside marks the 50% threshold.

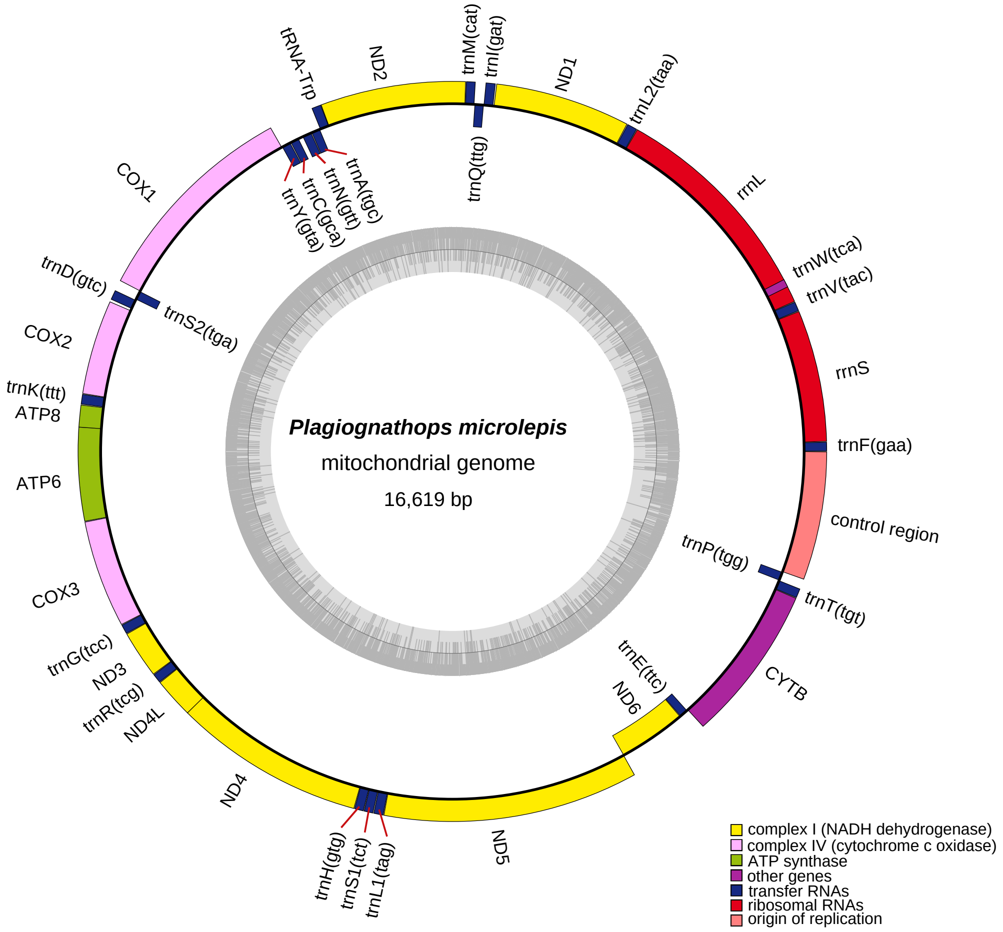

Figure S4. Circos diagram showing collinearity between the Haploid A and Haploid B.

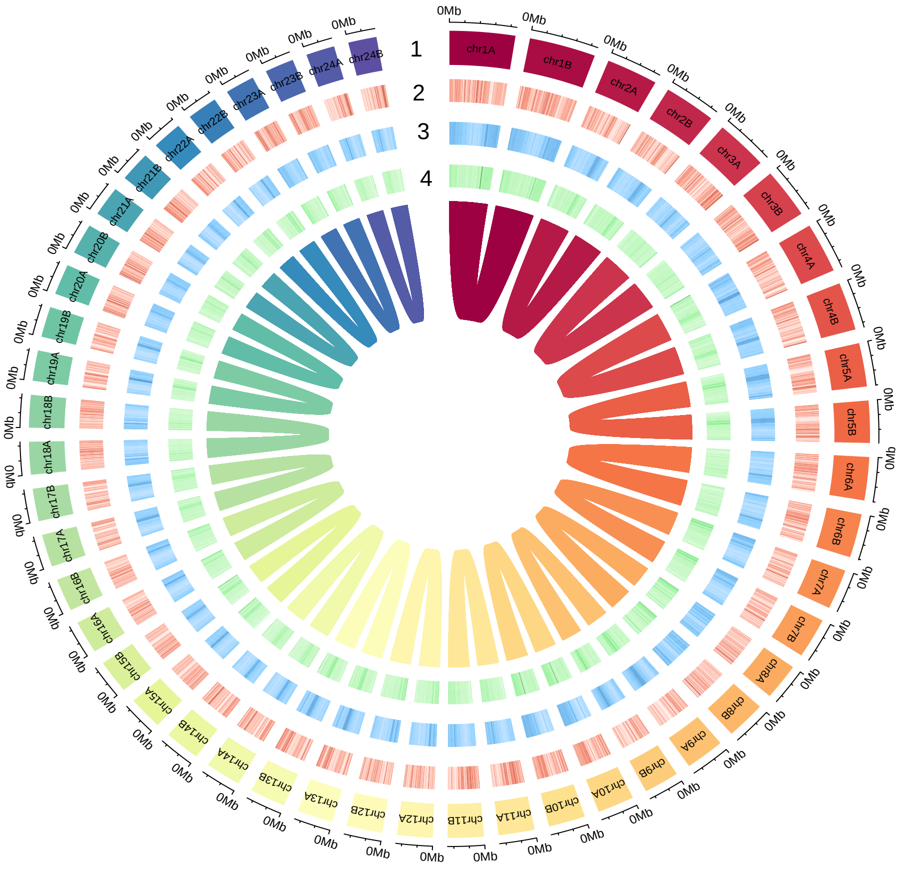

Figure S5. Comparison of the distribution characteristics for gene length (a), CDS length (b), exon length (c) and intron length (d) in the genomes of *P. microlepis* and 5 related species.

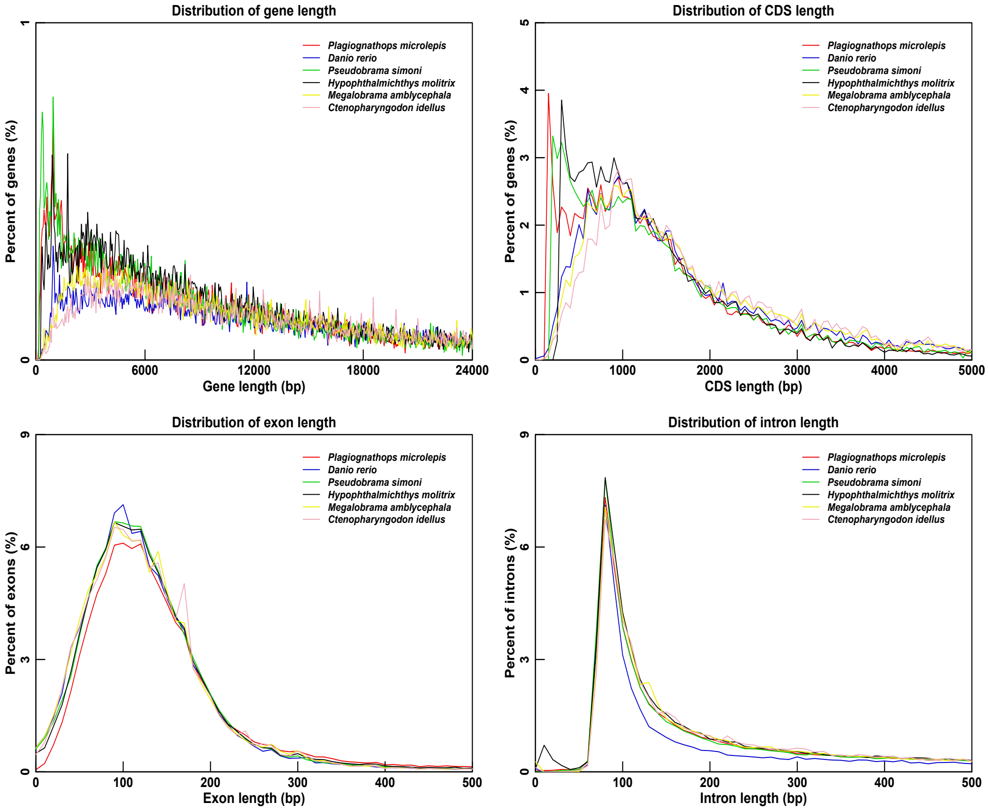

Supplement: Supplementary file 1 — Supplementary Figures [file 41597_2024_3645_MOESM1_ESM.pdf]
